# Characterization of porcine oocytes stained with Lissamine Green B and their developmental potential *in vitro*

**DOI:** 10.1590/1984-3143-AR2020-0533

**Published:** 2020-11-24

**Authors:** Alexandra Bartkova, Martin Morovic, Frantisek Strejcek, Matej Murin, Michal Benc, Florina Popovska Percinic, Jozef Laurincik

**Affiliations:** 1 Faculty of Natural Sciences, Constantine the Philosopher University in Nitra, Nitra, Slovak Republic; 2 Institute of Animal Physiology and Genetics, Czech Academy of Sciences, Libechov, Czech Republic; 3 Faculty of Veterinary Medicine, Ss. Cyril and Methodius University in Skopje, Skopje, Republic of North Macedonia

**Keywords:** pig, oocyte quality, Lissamine Green B, parthenogenetic activation, mass spectrometry

## Abstract

Traditional methods for the evaluation of oocyte quality are based on morphological classification of the follicle, cumulus-oocyte complex, polar body and meiotic spindle. This study is focused on the differences between the morphological assessment of oocyte quality, the assessment based on Lissamine Green B (LB) staining and the analysis of oocytes using a proteomic approach. We evaluated the effectiveness of electrochemical and chemical parthenogenetic activation under our laboratory conditions and evaluated the applicability of Lissamine Green B staining of cumulus-oocyte complexes (COCs) as a non-invasive method for predicting the maturational and developmental competence of porcine oocytes cultured *in vitro*. We determined that chemical parthenogenetic activation using ionomycin and 6-dimethylaminopurine was slightly more effective than electrochemical activation. After oocyte selection according to LB staining, we found significant differences (P<0.05) between the LB- group and LB+ group and the control group in their maturation, cleavage rate and rate of blastocysts. Proteomic analyses identified a selection of proteins that were differentially expressed in each group of analysed oocytes. Oocytes of the LB- group exhibited an increased variability of proteins involved in transcription regulation, proteosynthesis and the protein folding crucial for oocyte maturation and further embryonic development. These results found a better competence of LB- oocytes in maturation, cleavage and ability to reach the blastocyst stage.

## Introduction

Oocytes quality has a significant influence on the success of *in vitro* maturation (IVM), fertilization (IVF), embryo development and possible implantation (Goto et al., 1988; Sirard et al., 2006; Hoshino, 2018; Murin et al., 2019). Lower oocyte quality and occurrence of morphological anomalies are related a lower rate of IVM, IVF and IVC, which means, that oocyte quality is a key attribute of effective *in vitro* manipulations and treatment of infertility. This fact is a reason why oocyte collection is based on a strictly qualitative assessment of the current state of oocytes and embryos (Vassena et al., 2003; Krisher, 2004).

Oocyte quality can determine the probability of success of *in vitro* techniques. Nevertheless, objective and non-invasive methods used for evaluation of oocyte quality have been a problematic for decades. The right approach to evaluation can provide valuable information for the selection of oocytes with higher developmental competence and may maximize the success of embryo development (Wang and Sun, 2007; Mtango et al., 2008).

The most integrated and easiest way to evaluate oocyte quality is the morphological determination of the entire cumulus-oocytes complex (Combelles and Racowsky, 2005). Morphological evaluation is a traditional method based on morphological classification of the size and shape of cumulus-oocyte complexes (COCs), polar bodies (PBs), oolemma, *zona pellucida* and meiotic spindles (Sun and Nagai, 2003; Wang and Sun, 2007; Lasiene et al., 2009). Morphological evaluation provides valuable information for the selection of COCs with high quality, but in general, this technique is still subjective (Coticchio et al., 2004; Wang and Sun, 2007; Lasiene et al., 2009).

Various non-invasive methods for oocyte quality evaluation have been practiced and suggested. The most used non-invasive methods are for example propidium iodide or Brilliant cresyl blue staining (Murin et al., 2019). These methods have got a lot of positives and negatives. Due this fact, Lissamine Green B (LB) could be very elegant solution of these negatives. Lissamine Green B is a non-toxic and synthetic stain composed of an organic acid dye with two aminophenyl groups (Kim et al., 2009) and was already used for the selection of porcine oocytes (Dutta et al., 2016). This vital staining can detect cell damage, which means that LB diffuses through the damaged membrane. LB is also used in ophthalmology to evaluate the integrity of the ocular membrane. In reproductive medicine and embryotechnology, LB is a fast, cheap, and objective way to visualize membrane damage in oocytes used for embryo production *in vitro.*

Under *in vitro* conditions, meiotic division is reinitiated after *in vitro* fertilization or parthenogenetic activation. Nowadays, parthenogenetic activation is a modern tool, in which embryogenesis begins without previous sperm fertilization, which means that this method eliminates the influence of sperm and embryogenesis depends exclusively on the oocyte quality. The parthenogenetic activation of the oocyte can be initiated by various physical, mechanical, and chemical stimuli such as ethanol, ionomycin, ionophore, or direct electrical impulses (Alberio et al., 2001; Lane et al., 2002).

Electrochemical activation is based on pore formation in the oocyte membrane, which can increase levels of intracellular Ca^2+^ due to the influx of extracellular Ca^2+^ from the activation medium. Chemical activation is based on a combination of ionomycin and 6-DMAP (Alberio et al., 2001; Bing et al., 2001; Prochazka et al., 2011). Ionomycin can be characterized as a selective ionophore, which can bind intracellular Ca^2+^ and 6-DMAP as an inhibitor of maturation promoting factors (MPF) and mitogen-activated proteinkinase (MAPK) activity, which leads to the successful formation of parthenogenetic embryos (Machaty and Prather, 1998; Alberio et al., 2001; Bing et al., 2001; Yin et al., 2002; Prochazka et al., 2011).

The aim of this study was to determine the differences between two methods of the assessment of oocytes quality based on Lissamine Green B (LB) staining and the morphological evaluation. We decided to evaluate the effectiveness of electrochemical and chemical parthenogenetic activation and evaluated the applicability of LB staining of cumulus-oocyte complexes (COCs) as a non-invasive method for predicting the maturational and developmental competence of porcine oocytes cultured *in vitro*.

## Materials and methods

Chemicals, culture media and all supplements were purchased from Sigma Chemical Co., AppliChem unless otherwise stated. Media were freshly prepared, pre-warmed at 38.5 °C and filtrated with 0.2 µm Whatman membrane filter directly before use.

Our experiments were based on a comparison of two methods of parthenogenetic activation. After evaluating the success of parthenogenetic activation, we focused on comparison of the morphological assessment of oocyte quality and the vital assessment by Lissamine Green B staining, where we evaluated MII phase achieving, cleavage rate, blastocyst rate and proteomic profiling.

### Collection of oocytes

Porcine ovaries were collected at a local slaughterhouse. The ovaries were transported within one hour to the laboratory in thermos at 38 °C and washed three times in pre-warmed phosphate-buffered saline solution (PBS) supplemented with 0.4% (wt/vol) bovine serum albumin (BSA; Merck; Darmstadt, Germany), 0.34 mM glucose, 5.5 mM pyruvate, 50 IU/mL penicillin and 50 μg/mL streptomycin. Oocytes with follicular fluid were aspirated from ovarian follicles using a needle and 5 ml syringe. Aspirates were collected in 15 mL Falcon tubes and allowed to settle for 20 min at room temperature. 2/3 of the supernatant was gently removed and the sediment was used for COC collection under a Nikon SMZ800N (Tokyo, Japan, 45x magnification) stereomicroscope. Cumulus-oocytes complexes were recovered, collected and washed three times in PBS (Sun et al., 2004).

### *In vitro* maturation (IVM) of oocytes

After the evaluation of oocyte quality, COCs were washed three times in Dulbecco´s modified Eagle´s medium (DMEM supplemented with 10 IU/mL PMSG (Prospec, Israel) and hCG (Prospec, Israel), 50 ng/ mL EGF, 100 ng/mL IGF1 and 5 ng/ mL FGF). The DMEM medium was pre-warmed for 1 hour in a Petri dish. COCs were cultured and placed in groups of 30 in 500 µL DMEM, for 44 hours at 38.5 °C under 5% CO_2_ in air.

### Parthenogenetic activation of oocytes

After *in vitro* maturation, the collected oocytes were divided into two experimental groups and different methods of parthenogenetic activation were applied. The first experimental group of oocytes was activated electrochemically and the second chemically.

At the beginning of electrochemical activation, cumulus cells were removed with gentle pippeting with a 100 µL micropipette without hyaluronidase. Denuded oocytes were washed in mPBS and cultivated for 4 hours in TL-HEPES + Ca^+^ medium. After cultivation, the oocytes were washed in SOR 2 medium and activated with 38 V for 100 µs in an electroporator (CF-150/B Electro-Cell Manipulator, Hungary). Subsequently, electrically activated oocytes were washed three times in porcine zygote medium 3 (PZM3) with 6-dimethylaminopurine (6-DMAP, 2mM) and cultured in groups of 30 in 500 µL of PZM3 with 6-DMAP for 3 hours at 38.5 °C under 5% CO_2_ in air (Yoshioka et al., 2002).

Chemical activation was based on the combination of ionomycin and 6-DMAP. Oocytes were denuded by gentle pipetting with a 100 µL micropipette. After that, oocytes were washed twice in mPBS and activated in groups of 30 for 5 min. with 5 µM ionomycin. The activated oocytes were washed three times in the PZM3 medium with 2mM 6-DMAP and cultivated for 5 hours. After cultivation, oocytes were washed three times and cultured in the same medium for 5 hours at 38.5 °C under 5% CO_2_ in air (Prochazka et al., 2011).

### *In vitro* cultivation

The activated oocytes were cultured in 50 µL drops of PZM3 overlaid with mineral oil for 7 days at 38. 5 °C with 5% CO_2_ in air. Cleavage rate was evaluated 48h after activation and blastocyst rate was observed after 168h of *in vitro* cultivation. (Yoshioka et al., 2002).

### Evaluation of oocyte quality

After the comparison of parthenogenetic activations, we decided for chemical activation in our subsequent experiments, where COCs were evaluated using morphological criteria and LB staining.

#### Morphological evaluation

For further study only high-quality COCs with a homogenous, transparent and un-fragmented ooplasm with more than two compact layers of cumulus cells were selected. The selected oocytes were divided into two groups, those selected by the morphological evaluation (control group) and those selected by the LB staining evaluation.

#### LB evaluation

**Figure 1:**
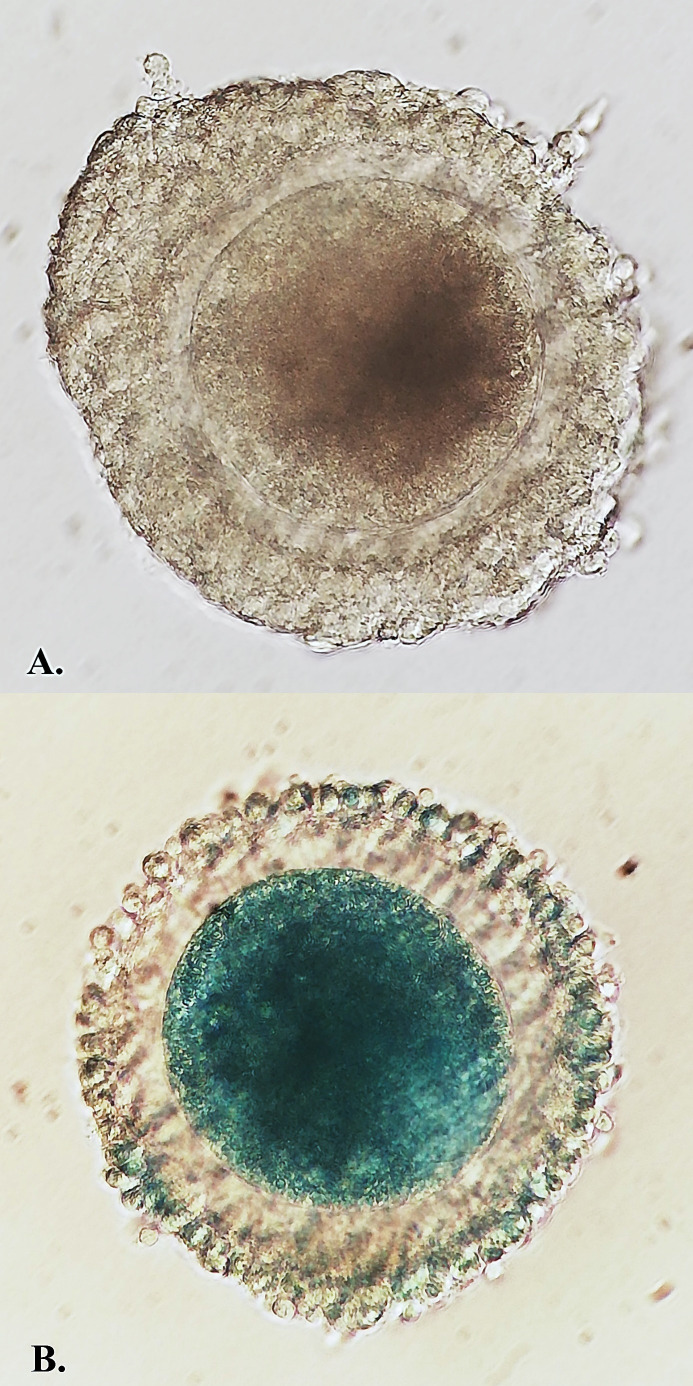
Classification of porcine oocytes after Lissamine Green B staining. (A) There is LB- oocyte, which represents high quality group; (B) There is LB+ oocyte, which represents low quality group (magnification X 400).

The selected COCs were placed in groups of 30 for 10 min. in 500 µL 0.5% LB at RT. Stained COCs were washed three times in mPBS (PBS + 0.4% bovine saline albumin-BSA) and observed under a Nikon SMZ800N stereomicroscope. After washing, the COCs were divided into two qualitative subgroups based on LB staining. We focused on detection of damaged cytoplasmic membrane, which was characterized by green stained ooplasm. Unstained (LB-) COCs with 0% stained cumulus cells were classified as high-quality COCs and stained (LB+) COCs with green ooplasm were designated as COCs with poor quality. In the LB+ group percentage of stained cumulus cells was not considered as an important marker (Figure 1) (Dutta et al., 2016).

### Sample preparation for proteomic profiling

For the nano HPLC-Chip-MS/MS analysis, samples of 20 LB- and LB+ oocytes were lysed in 200 μL of SDT lysis buffer containing 4% SDS (w/v), 0.1 M DTT, 0.1 M Tris/HCl pH 7.6 and protease inhibitors (1x Roche complete) by shaking for 1 hour at 37 °C. Immediately after lysis, samples were cooled down to 4 °C and centrifuged at 16 000 x g for 5 min. The supernatant was transferred to 3 kDa molecular weight cut-off filter plates (Amicon Ultra-0.5 ml 3K, Millipore) and spun at 14 000 x g for 40 min. For the extraction of tryptic peptides from the complex protein mixture, a modified filter-aided sample preparation (FASP) protocol was used according to Wisniewski et al. (2009). Detergent removal by buffer exchange was performed in two sequential washes of filter plates with 200 µL of 8 M urea in 0.1 M Tris/HCl pH 8.5 (UA), each followed by centrifugation at 14 000 x g for 40 min. Flow-through was removed from the collection tubes. Proteins were then alkylated in 100 µL of 0.05 M iodoacetamide in UA by mixing at 600 rpm in a thermo-mixer for 1 min and incubating without mixing for 20 min. After centrifugation at 14 000 x g for 30 min, two additional washes using 100 µL of 0.05 M NH_4_HCO_3_, with 10 min at 14 000 x g after each wash, were included to remove excess urea and prepare the proteins for digestion. Protein digestion was performed by adding 2 µg of trypsin (Trypsin Gold, Promega) in 0.05 M NH_4_HCO_3_ and incubating at 37 °C overnight. Peptides were recovered by spinning the filter plates upside down at 14 000 x g for 40 min. The mixture was vacuum dried and the pellet was resuspended in 50 µL of mobile phase (97% water, 3% acetonitrile).

### Protein identification by tandem mass spectrometry (nano HPLC-chip-MS/MS)

The tryptic peptides were loaded into the 40 nL enrichment column (filled with Zorbax SB C18, 5 μm) of an Agilent 1260 ChipCube MS Interface by an Agilent 1260 Capillary Pump (Agilent Technologies, Palo Alto, USA). After loading and desalting of the sample in the chip enrichment column peptides were eluted in a forward flush and transferred to the analytical column at a flow rate of 600 nL/min by an Agilent 1260 Nano pump with an increasing percentage of organic phase. The mobile phase consisted of an aqueous (A) or acetonitrile (B) solution of formic acid (0.1%, v/v). The chromatographic separation was achieved with a gradient elution using the following sequence: 0 min, 3% B; 2 min, 3% B; 25 min, 50% B; 30 min, 50% B; 35 min, 95% B; 40 min, 95% B; 45 min, 3% B followed by a post-period of 10 min for column re-equilibration. The analytical column was connected to a Q-TOF Agilent 6500 Series mass spectrometer. A 1750V tension was applied to the electrodes of the nanospray ionization chamber. High-purity nitrogen (99.99999%) was used as the collision gas and the collision energy was settled as a function of the precursor ion mass and charge. MS/MS spectra were acquired by automatic switching between MS and MS/MS mode (auto MS/MS mode). Acquired MS/MS data were analyzed using the SpectrumMill search engine (Agilent Technologies, Palo Alto, USA). Database searches were performed against the self-built database from the UniProt porcine proteome (UP000008227). The following autovalidation criteria of SpectrumMill software were used to validate the identified proteins and peptides: the minimum score for proteins was 10, minimum scores for spectra resulting from fragmentation of 2+, 3+, and 4+ precursor ions were 8, 7 and 9, respectively, with a scored peak intensity value of at least 60%.

### Assessment of *in vitro* oocyte maturation

After 48 hours of *in vitro* maturation, cumulus cells were removed from oocytes. We prepared microscope slides, where two strips of grease were placed approximately 1.5 cm apart. The oocytes were transferred to the space between strips, covered with a cover glass and fixed for 24 hours in a 3:1 mixture of ethanol (96%) and acetic acid. After fixation, the oocytes were stained with lacmoid and the success rate of *in vitro* maturation was evaluated with phase-contrast microscope (Laurincik et al., 1994).

### Statistical analysis

Our data were expressed as means ± standard deviation of the mean (SD). We evaluated our data by one-way analysis of variance (ANOVA single factor) and t-test (SigmaPlot 12.0, London, UK), where a probability of <0.05 was considered to be statistically significant.

### Ethical standards

The authors assert that all procedures contributing to this work comply with the ethical standards of the relevant national and institutional guides on the care and use of laboratory animals.

## Results

The COCs obtained from the local slaughterhouse were divided into several experimental groups and used for our experiments, which based on the comparison of different techniques of parthenogenetic activation, LB staining and proteomic profiling.

### *In vitro* maturation

In total 268 COCs were divided into 3 experimental groups: the control group (CG), LB+ group and LB- group. The evaluation of proper *in vitro* maturation was based on the presence of condensed chromosomes in an equatorial position. The maturation rate of the control group, which consisted of morphologically graded COCs, was 74.03%. In the LB+ group it was 22.80% and in LB- it was 82.24%. These data showed significant differences between the control group and LB+ group and between the LB+ and LB- groups (Table 1).

**Table 1:** Maturation ability of porcine oocytes treated with LB *in vitro.* Five replicates of this experiment were performed. In brackets, numbers of oocytes are stated. Within a column, values with different small letters (a, b) are significantly different with P<0.05.

Experimental group	No. of oocytes	% of oocytes in MII
Control	104	74.03 ± 6.18 (77)^a^
LB+	57	22.80 ± 8.03 (13)^b^
LB-	107	82.24 ± 1.02 (88)^a^

### Proteomic evaluation of LB- and LB+ oocytes

**Figure 2:**
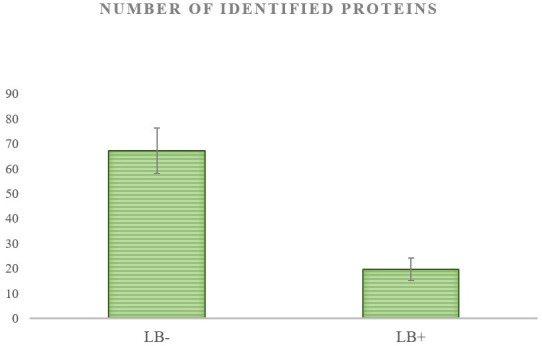
Differences in number of identified proteins from 20 LB- and LB+ oocytes with significance at level P<0.05 for three independent proteomic analyses (sample preparation and protein identification by tandem mass spectrometry).

In this part of the study, we compared the protein patterns of LB- and LB+ pig oocytes using the off-gel approach in combination with tandem mass spectrometry. Based on the morphological observations (chromosome condensation) and Lissamine Green B staining, the oocytes were divided into two different qualitative groups where LB- oocytes were considered to be the high-quality oocytes with the highest maturation rate (82%). Despite difficulties with obtaining a sufficient amount of material to identify low-abundance proteins by mass spectrometry, we were able to unambiguously discern the differences (P<0.05) between the proteomic profiles of LB- and LB+ oocytes. In the LB- oocytes, we were able to identify 67 proteins, whereas in the LB+ group we could identify only 19 proteins (Figure 2). Our proteomic analysis also identified a selection of proteins that appeared to be differentially expressed in each group of analysed oocytes. Oocytes of the LB- group exhibited an increased variability of proteins involved in RNA pol II- and RNA pol III-mediated transcription regulation, proteosynthesis and protein folding. LB+ oocytes lacked the majority of transcription-related proteins and mostly contained enzymes or proteins from the *zona pellucida* (Table 2).

**Table 2:** List of 25 selected proteins from Spectrum Mill – Protein peptide/Summary involved in fertilization, transcription and translation processes sorted by function/activity.

*Protein function*	*Accession number* ^*^	*Protein name*	*MW (kDa)*	LB- oocytes	LB+ oocytes
***zona pellucida* proteins**	P42099	*Zona pellucida* sperm-binding protein 2	79,67	**+**	**+**
	P42098	*Zona pellucida* sperm-binding protein 3	46,20	**+**	**+**
	Q07287	*Zona pellucida* sperm-binding protein 4	59,29	**+**	**+**
**enzymatic activity**	A0A287A1A8	Acyl-coenzyme A oxidase	66,47	**+**	**+**
	F1SUQ9	Protein-arginine deiminase type-6	76,87	**+**	**+**
	I3LBD5	Serine/threonine-protein kinase 10	112,01	**+**	**+**
	Q9GL50	Metalloreductase STEAP1	39,89	**+**	**-**
	F1SAN1	Caspase 14, apoptosis-related cysteine peptidase	27,70	**+**	**-**
**RNA Pol II and RNA Pol III regulation activity**	F2Z5J6	Transcription initiation factor IIB	34,81	**+**	**-**
	F1SPQ0	DNA-directed RNA polymerase subunit beta	122,58	**+**	**-**
	A0A287BRQ3	ETS domain-containing protein	55,80	**+**	**-**
	F1RUB7	Transcriptional regulator Kaiso	74,24	**+**	**-**
	A0A286ZZH2	Storkhead box 1	110,23	**+**	**+**
	A6N8P5	Multiprotein bridging factor 1	16,38	**+**	**-**
	A0A287B954	Transcriptional repressor p66-beta isoform X1	65,21	**+**	**-**
	I3L8J9	Zinc finger protein 77	58,76	**+**	**+**
	A0A286ZMX7	ANK_REP_REGION domain-containing protein	116,67	**+**	**-**
	A0A286ZJG5	GYF domain-containing protein	151,40	**+**	**-**
	I3LLE2	GLTSCR1 domain-containing protein	147,02	**+**	**-**
**chaperone activity, protein folding, proteosyntesis**	P63053	Ubiquitin-60S ribosomal protein L40	14,71	**+**	**+**
	I3LQ79	Major vault protein	99,64	**+**	**+**
	F1SL22	von Willebrand factor	321,78	**+**	**+**
	A0A287BIL8	Endoplasmic reticulum chaperone BiP	72,22	**+**	**-**
	A0A287AQK7	Heat shock protein HSP 90-alpha	90,58	**+**	**-**
	A0A286ZK74	RRM domain-containing protein	25,28	**+**	**-**

* Protein accession number referring to UniProt database. **+** detected, - undetected.

### Electrochemical activation vs. chemical activation

This experiment was carried out to compare electrochemical and chemical activation. In the first experimental group, activated by electrochemical activation (n=147), the cleavage rate of embryos was 76.87% (n=113) and rate of blastocyst formation was 12.34% (n=14). In the second experimental group activated by chemical activation (n=263), the cleavage rate was 79.85% (n=210) and rate of blastocyst formation was 17.14% (n=36). Although we observed small difference between two techniques of parthenogenetic oocyte activation, chemical activation was slightly more effective than electrochemical approach under our laboratory conditions, so we decided to utilise the chemical activation of LB groups (Table 3).

**Table 3:** Parthenogenetic activation of porcine oocytes after electrochemical and chemical activation. Five replicates of this experiment were performed. In brackets, numbers of oocytes or embryos are stated. Within a column, values with different small letter (a) are non-significally different with P<0.05.

Experimental group	No. of oocytes	% of embryos development	
		Cleavage rate	Blastocysts rate
Electrochemical activation	147	76.87±2.90 (113)^a^	12.34 ± 2.09 (14)^a^
Chemical activation	263	79.85 ± 3.48 (210)^a^	17.14 ± 1.06 (36)^a^

### Effect of vital staining during embryogenesis

The cleavage rate of the control group was 80.20% (n=158), in the LB+ group it was 16.66% (n=12) and in the LB- group it was 84.32% (n=258). Significant differences were observed between the LB- group and LB+ group (P<0.05) and also between the control group and LB+ group (P<0.05). The highest blastocyst developmental rate was detected in the LB- group, where it was 28.68% (n=74). In the control group, the blastocyst rate was 20.89% (n=33) and in the LB+ group it was 8.33% (n=1) (Table 4). A significant difference was identified between the LB- group and LB+ group (P<0.05) and between the LB+ group and the control group (P<0.05).

**Table 4:** Chemical activation of porcine oocytes treated with LB *in vitro*. Five replicates of this experiment were performed. In brackets, numbers of oocytes or embryos are stated. Within a column, values with different small letters (a, b) are significantly different with P<0.05.

Experimental group	No. of oocytes	% of embryos development	
		Cleavage rate	Blastocysts rate
Control	197	80.20 ± 2.83 (158)^a^	20.89 ± 2.44 (33)^a^
LB+	72	16.66 ± 4.92 (12)^b^	8.33 ± 8.25 (1)^b^
LB-	306	84.32 ± 0.91 (258)^a^	28.68 ± 1.72 (74)^a^

## Discussion

Oocyte quality is one of the key factors, which influences the success of embryo production *in vitro*. Oocyte quality can be characterized as a summary of individual and comprehensive assessments.

In this study, the determination of oocyte quality was done by LB staining as a non-invasive selective method detecting the cells and membrane damage of porcine oocytes before *in vitro* maturation. LB was used back in 1990 for the detection of damage to reticuloendothelial cells. In 2000, Kim et al. (2009) used LB for the *in vitro* staining of rabbit and human corneal epithelial cells and in 2015, Dutta et al. (2016) used LB for *in vitro* staining of porcine COC´s. Based on these applications, we decided to test the effect of this non-toxic and non-carcinogenic dye on the *in vitro* maturation of oocytes and development of porcine embryos.

Our study is based on the comparison of the LB staining method with the routine morphological evaluation of oocytes and more comprehensive proteomic profiling. Our observations indicate that the evaluation of the oocytes based on LB staining is more effective than the morphological evaluation of oocyte quality. Under our laboratory conditions, we managed to optimize the cultivation and maturation of porcine oocytes. Our results show that we achieved a 74.03% maturation success rate in the control group. In comparison with the control group, we can conclude that the LB- group achieved a higher maturation rate (82.24%) than both the control group (74.03%) and LB+ group (22.80%). Our results can be compared with Dutta et al. (2016), who focused on the evaluation of cumulus cells by Lissamine Green B. In Dutta et al. (2016) successful maturation rate was reached 79.9% in A grade group, which is very similar to our control group (74.03%). With porcine A grade oocytes (high quality oocytes after LB staining), the successful maturation rate reached 84.70%, which is almost identical to our LB- oocytes (82.24%). Their study also observed a higher competence of C grade group (lower quality after LB staining) to reach MII (62.26%), which is significantly different as our result in LB+ group (22.80%). In Dutta et al. (2016), very similar differences were observed after parthenogenetic activation too. Based on the results, we can say that the detection of membrane damage leads to a significant reduction in oocyte developmental potential, which is more important than the detection of damaged cumulus cells.

Vital selection based on LB staining can be compared with vital selection by Brilliant cresyl blue (BCB) staining. With porcine BCB+ oocytes (high quality), Egerszegi et al. (2010) reported that after 44 hours of *in vitro* cultivation, the successful maturation rate reached 81.6%, which is almost identical to our LB- oocytes. Their study also observed a significantly higher competence of BCB+ oocytes to reach MII than BCB- oocytes, which also correlates with our results (LB- 82.24% vs. LB+ 22.80%).

Additionally, the results achieved by LB staining fully correspond with our proteomic evaluation of porcine oocytes. Motivated by relatively weak knowledge about mammalian oocyte maturation in this field of research, we decided to utilise a proteomic approach to identify proteins that are differentially expressed during porcine oocyte maturation and thus support our preceding results (LB staining). Due to the lack of sufficient biological material (LB+ oocytes), the off-gel proteomic approach was applied, in which the whole protein extract without any further separation was analysed by tandem mass spectrometry. Also, a unique LC-based nano HPLC-chip workflow was followed to minimize the loss of analysed material. In the complete list of identified proteins isolated from LB- (n=67) and LB+ (n=19) oocytes, we selected 25 proteins important for fertilization, transcription, translation and post-translational (protein folding) processes. Except for *zona pellucida* sperm-binding proteins (2, 3 and 4), crucial for sperm binding, induction of the acrosome reaction and preventing post-fertilization polyspermy, we observed that oocytes of the LB- group exhibit an increased variability of proteins (n=22) involved in gene expression processes compare to LB+ oocytes (n=6). With the LB+ oocytes, we may suppose that these absent proteins were not expressed in oocytes at the time of evaluation or more likely that their amounts were under the detection limit of the instruments we used. These differences in the protein composition of qualitatively distinct oocytes (LB- vs. LB+ oocytes) correspond to those observed by Ellederova et al. (2004) in porcine GV, MI and MII stage oocytes. Based on the assumption that the quality and thus the fertilization competence of oocytes rises from the GV to the MII stage, the comparative proteomic approach was used to analyze the oocytes at different stages of maturation to characterize the candidate proteins which were differentially expressed during *in vitro* maturation. The results of their proteomic evaluation of porcine oocytes showed that some of the identified proteins were more abundantly expressed in high-quality MII-stage oocytes than in GV oocytes. Similar results were achieved by the team of Vitale et al. (2007) and Virant-Klun et al. (2016), where the protein profiles of oocytes (mouse, human) during *in vitro* maturation were studied. In both cases, the proteomic analysis of GV and MII-stage oocytes identified a selection of unique proteins that appeared to be differently regulated during oocyte maturation.

We achieved a high cleavage rate in both types of parthenogenetic activations, but the rates of blastocysts were different. Our results indicated that better embryo development was obtained via chemical activation with ionomycin and 6-DMAP. A similar result was observed by Che et al. (2007) (71% cleavage vs. 32% blastocysts). Prochazka et al. (2011) reported a successful cleavage rate 73% and blastocyst rate of 38%, which is similar to our results. Their study also confirmed the beneficial effect and high rate of blastocysts obtained by the combination of ionomycin and 6-DMAP.

With the chemical activation of LB oocytes, our data showed significant differences between the LB+ and LB- group. *In vitro* maturation and *in vitro* parthenogenetic activation of LB- oocytes were more successful than the LB+ group.

## Conclusions

In conclusion, our study indicates that chemical activation combined with LB staining can result in effective production of parthenogenetic porcine embryos. The evaluation of the oocytes quality based on Lissamine Green B staining is vital, cheap and quick selecting method and it is slightly more probative than the morphological evaluation of oocyte quality. From the proteomics point of view, the differentially regulated proteins acting during oocyte *in vitro* maturation may represent a group of promising biomarkers of oocyte maturation and quality.
